# Google searches do not correlate with melanoma incidence in majority English speaking countries

**DOI:** 10.1038/s41746-018-0050-4

**Published:** 2018-09-05

**Authors:** Laura McDonald, Alex Simpson, Sophie Graham, Anna Schultze, Beth Nordstrom, Piyush Durani, Faisal Mehmud, Sreeram V. Ramagopalan

**Affiliations:** 1 Centre for Observational Research and Data Sciences, Bristol-Myers Squibb, Uxbridge, UB8 1DH UK; 2 Real-World Evidence, Evidera, London, W6 8DL UK; 3 Real-World Evidence, Evidera, Waltham, MA 02541 USA; 4 Bristol-Myers Squibb, Uxbridge, UB8 1DH UK

**Keywords:** Cancer epidemiology, Epidemiology

## Abstract

Recent reports have suggested that internet search behaviour may be a valuable tool to estimate melanoma incidence and mortality. Previous studies have used incorrect statistical methods, were focussed on the United States and/or did not use non-cancer control search terms to provide a context for interpreting the effects seen with the cancer-related terms. Using more robust statistical methods we found that no cancer search terms were significantly, or strongly correlated with melanoma incidence in 6 countries.

## Introduction

The internet is an important source of health information for patients. In 2013 surveys showed that >70% of adults looked up online health information in the preceding year.^1^ Although many of these online health searches may be more general or related to an already-diagnosed condition or planned treatment, 35% of Americans reported looking online specifically to determine what medical condition they may have.^1^ Google is the world’s most popular search engine^2^ and ~5% of all Google searches are for health-related information.^3^ This vast volume of search data is therefore a potential option to provide a robust and real-time surveillance system of epidemics and diseases.^4^

To this end, recent reports have suggested that Google internet search behaviour may be a valuable tool to estimate cancer incidence and/or mortality, particularly when national registry data are unavailable.^5,6^ Most of these studies, however, have been focussed largely on the United States (US).^5–7^ Further, some studies have used potentially inappropriate statistical methods,^5,6^ neglecting to consider the auto-correlated nature of the time series search data (i.e., the dependency of observed search activity on previous search activity).^8^ One such study found that the search term “skin cancer”, but not “melanoma”, was correlated with melanoma mortality, but not incidence in the US.^5^ Another study, also limited to the US and not correcting for autocorrelation, found the “melanoma” search term to be weakly associated with both melanoma incidence and mortality.^6^ Without appropriate control terms independent from cancer terms, the correlations observed in both cases could be spurious, as any number of other non-cancer related terms might have also demonstrated a similar correlation (for example, Google searches in general show seasonal patterns that would correlate generically with multiple phenomena with the same seasonal pattern). In order to address the concerns raised with previous studies, we sought to examine correlations of internet search behaviour and melanoma incidence in the US and other majority native English-speaking countries, accounting for auto-correlation in our analysis as well as assessing the relative specificity of cancer search terms.

## Results

Correlations of SVI and melanoma incidence in the US states between 2011 and 2014 are shown in Table 1. Overall, no search terms were strongly correlated with melanoma incidence. The strongest correlation observed was 0.31 (95% confidence interval (CI), −0.19 to 0.43) with “Melanoma”. None of the correlations achieved statistical significance.

**Table 1:** Correlation of cancer and control search terms against melanoma incidence in US states between 2011-2014

	Cancer term		Control term	
	Skin cancer	Melanoma	European Union	Google
Incidence	−0.049	0.313	−0.004	0.117
95% CI	(−0.298, 0.336)	(−0.190, 0.434)	(−0.424, 0.592)	(−0.267, 0.507)
*p*-value	0.485	0.596	0.499	0.535

Correlations of SVI over time with melanoma incidence in English speaking countries is shown in Table 2. The strongest correlation was observed with the “Melanoma” search term (0.52 (95% CI, −0.43 to 0.89)) and again, none of the correlations achieved statistical significance.

**Table 2:** Correlation of cancer and control search terms against melanoma incidence for English speaking countries (England, Republic of Ireland, Australia, New Zealand, Canada) between 2004 and 2016

	Cancer term		Control term	
	Skin cancer	Melanoma	European Union	Google
Incidence	−0.032	0.523	−0.028	0.207
95% CI	(−0.657, 0.709)	(−0.434, 0.887)	(−0.389, 0.205)	(−0.0130, 0.480)
*p*-value	0.981	0.662	0.983	0.874

## Discussion

In this study we found that no Google search terms we tested were significantly, or strongly correlated with melanoma incidence in 6 countries. Using a mixed model, we have accounted for the auto-correlation in the time series search data and by including control search terms, we were able to assess inferential relationships between search terms and melanoma incidence. Indeed, given that all of the results were non-significant, we were unable to confirm previously published findings presented by Wehner et al.,^6^ however, these results do corroborate those of Bloom et al.^5^ in that at the US state level, skin cancer/melanoma searches do not correlate with melanoma incidence. Such differences might have occurred in the former case due to a differing time period used; Google SVI is standardised to the maximum value of search volume in the time period considered, meaning that for different but overlapping time periods, the relative overlapping values for SVI can be drastically different.^9^ It has been suggested that internet search data may be particularly useful where national registry data on cancer are unavailable. As we have found here that there was no strong or significant association between search terms and cancer incidence, using search data as a proxy for estimating cancer incidence therefore appears unwarranted.

As cancer incidence trends are relatively slow to change, Google search data may be more useful for tracking epidemic conditions where incidence can dramatically change over a few weeks or months. The Google flu trend prediction tool was developed on this premise,^4^ but was discontinued as correlation of search terms with flu incidence were not causally related and as such when internet search patterns changed temporally, the flu trend prediction tool was not able to accurately predict disease occurrence.^4^ Improvements to infectious disease prediction have come when search data is combined with traditional surveillance data.^10^

In summary, whilst the public health benefits of being able to track disease incidence using internet search volumes would be significant, such as for epidemic prediction and surveillance, more thought is likely needed to do this with rigour to enable robust insights to be obtained.

## Methods

Age-adjusted melanoma incidence averaged for males and females was obtained for the US for 2011–2014,^11^ England^12^ for 2004–16, New Zealand^13^ for 2004–15, Australia^14^ for 2004–13, Canada^15^ for 2004–16 and the Republic of Ireland for 2004–14.^16^ Google Trends^17^ quantifies interest in topics at the population level by analysing all search queries for a specific term. Search volume indexes (SVIs) are normalized values based on total searches during a specified period per selected region. We obtained SVIs for each US state or non-US country from January 1, 2004, to January 1, 2017, for the search terms “melanoma” and “skin cancer” (representing a lay term for melanoma). Control search terms were “Google” and “European Union” and SVIs for these were obtained for the same time periods. Control search terms were selected by the authors to determine if unrelated terms might also show similar correlations to the cancer related search terms, to this end the control terms were utilised in exactly the same way as the cancer related search terms. For the US state model, annualised SVIs were available directly from Google with each state contributing a data point for each year. For the English-speaking countries model, we retrieved annual SVI data for each term that was stratified by month for each country and calculated the average of these 12 monthly values to create an annual estimate in order to match the annual cancer incidence data. Due to the autocorrelation within these values, we derived a mixed model to calculate a correlation coefficient between cancer incidence and SVIs over time. An unstructured covariance matrix was used initially in the models as per Hamlett et al.^18^ However, in cases where convergence was not achieved and would not produce viable estimates, we used an autoregressive structure as the next best choice. Bootstrapped 95% confidence limits for the correlation coefficients were estimated and *p*-values obtained from an asymptotic Fisher test. All analysis was performed using SAS version 9.4 (SAS Institute Inc., Cary, North Carolina).

### Code availability statement

SAS version 9.4 was used for all analyses.

## Data Availability

The datasets analysed during the current study are publicly available with websites cited for all data.
